# Coexistence of Bilateral Eales’ Disease and Serpiginous-Like Choroiditis in a QuantiFERON-Positive Patient

**DOI:** 10.22336/rjo.2026.63

**Published:** 2026

**Authors:** Mustafa Kayabaşı, Ömer Kartı, Ziya Ayhan, Eyüp Sabri Uçan, Ali Osman Saatci

**Affiliations:** 1 Department of Ophthalmology, Balıkesir Atatürk City Hospital, Balıkesir, Turkey; 2 Department of Ophthalmology, Dokuz Eylül University, İzmir, Turkey; 3 Department of Pulmonary Diseases, Private Practice, İzmir, Turkey

**Keywords:** Eales’ disease, multimodal imaging, serpiginous-like choroiditis, tuberculosis, vitreous hemorrhage, SLC = Serpiginous-like choroiditis, VEGF = Vascular endothelial growth factor

## Abstract

**Objective:**

To describe an unusual case of concurrent bilateral Eales’ disease and serpiginous-like choroiditis in a young immunocompetent woman with a positive interferon-gamma release assay test in the absence of clinically active systemic tuberculosis.

**Methods:**

Retrospective case report.

**Results:**

We evaluated a 25-year-old immunocompetent woman with left visual decline. She experienced an episode of left visual loss that improved spontaneously almost six months earlier. Slit-lamp examination showed no uveitis-related anterior segment findings bilaterally. Fundoscopy yielded almost symmetric serpiginous-like scars in the upper temporal quadrant. There was temporal peripheral periphlebitis in both eyes, together with right retinal neovascularization and a left temporal fibrotic-looking ridge and intravitreal hemorrhage. Fluorescein angiography and wide-field optical coherence tomography angiography showed peripheral nonperfusion and right retinal neovascularization. We performed systemic investigations and laboratory tests. QuantiFERON-TB Gold^®^ test (Cellestis Inc., Carnegie, Victoria, Australia) was positive. The patient and her computed tomography chest scan were reviewed by an experienced pulmonary specialist, who did not confirm any sign of active tuberculosis. Our diagnosis was bilateral serpiginous-like choroiditis and Eales’ disease. We treated the left eye with a single intravitreal ranibizumab injection followed by scattered laser photocoagulation, while the right eye received scatter laser treatment only.

**Discussion:**

The rare coexistence of Eales’ disease and serpiginous-like choroiditis in a QuantiFERON-positive patient without any evidence of active systemic tuberculosis may represent distinct ocular manifestations of a shared tuberculosis-associated immune-mediated process. Multimodal imaging is essential for recognizing this unusual overlap.

**Conclusions:**

This case shows that tuberculosis-associated immune mechanisms may cause different clinical manifestations, and very rarely, Eales’ disease and serpiginous-like choroiditis may occur simultaneously.

## Introduction

Eales’ disease is an idiopathic inflammatory vaso-occlusive disorder characterized by peripheral perivascular phlebitis, areas of capillary non-perfusion, and retinal neovascularization, predominantly affecting young male individuals [1]. The clinical spectrum includes recurrent vitreous hemorrhage, epiretinal membrane formation, macular edema, subretinal fibrosis, and tractional complications arising from the proliferative nature of the disease [2]. Although its precise etiology remains unclear, the current evidence suggests that the disease may represent a hypersensitivity reaction to *Mycobacterium tuberculosis (M. tuberculosis) *antigens [3].

Serpiginous-like choroiditis (SLC) is widely recognized as a manifestation of tubercular uveitis and differs from serpiginous choroiditis with its multifocal distribution, clinical course, and therapeutic response [4]. Unlike serpiginous choroiditis, SLC is considered to arise from an infectious or para-infectious, immune-mediated process triggered by mycobacterial antigens [5].

Although both Eales’ disease and SLC have individually been linked to tuberculosis-related immune mechanisms [1,4], their coexistence in the same individual is distinctly uncommon. Here, we report an unusual case of concurrent Eales’ disease and SLC in a patient with a positive QuantiFERON-TB Gold^®^ test (Cellestis Inc., Carnegie, Victoria, Australia), without evidence of active systemic tuberculosis, underscoring the diagnostic challenges and therapeutic considerations associated with this rare overlap.

## Case report

A 25-year-old immunocompetent overweight woman presented with a 10-day history of visual decline in her left eye. She described a prior episode of vision loss in her left eye six months earlier, lasting approximately one month, that resolved without any treatment. Her medical history was unremarkable except for asymptomatic polycystic ovary syndrome.

Her best-corrected Snellen visual acuity was 20/20 in the right eye and 20/200 in the left eye. Slit-lamp examination findings were unremarkable, and intraocular pressure was 16 mmHg in both eyes. On right fundoscopy, paramacular, irregular, yellowish-white, placoid chorioretinal lesions with indistinct borders were present superotemporally, along with perivascular sheathing of the retinal vessels at the temporal periphery. In the left eye, similar irregular, yellowish-white, placoid chorioretinal lesions with indistinct borders were also observed superior to the fovea, accompanied by mild to moderate vitreous hemorrhage and a vertical fibrotic ridge at the temporal peripheral retina. Blue fundus autofluorescence imaging (Heidelberg Spectralis^®^, Heidelberg Engineering, Heidelberg, Germany) demonstrated areas of hypoautofluorescence corresponding to the clinically apparent placoid chorioretinal lesions in both eyes (**Fig. 1A, B**). Fluorescein angiography (Heidelberg Spectralis^®^, Heidelberg Engineering, Heidelberg, Germany) of the right eye revealed late hyperfluorescence of the placoid lesions consistent with fluorescein staining, along with retinal neovascularization, increased vascular tortuosity, peripheral capillary nonperfusion, and retinal neovascularization in the temporal periphery (**Fig. 1C**). In the left eye, although visualization was partially obscured by vitreous hemorrhage; similar late hyperfluorescence of the serpiginous-like lesions was observed, along with areas of capillary nonperfusion in the temporal periphery (**Fig. 1D**).

**Fig. 1:**
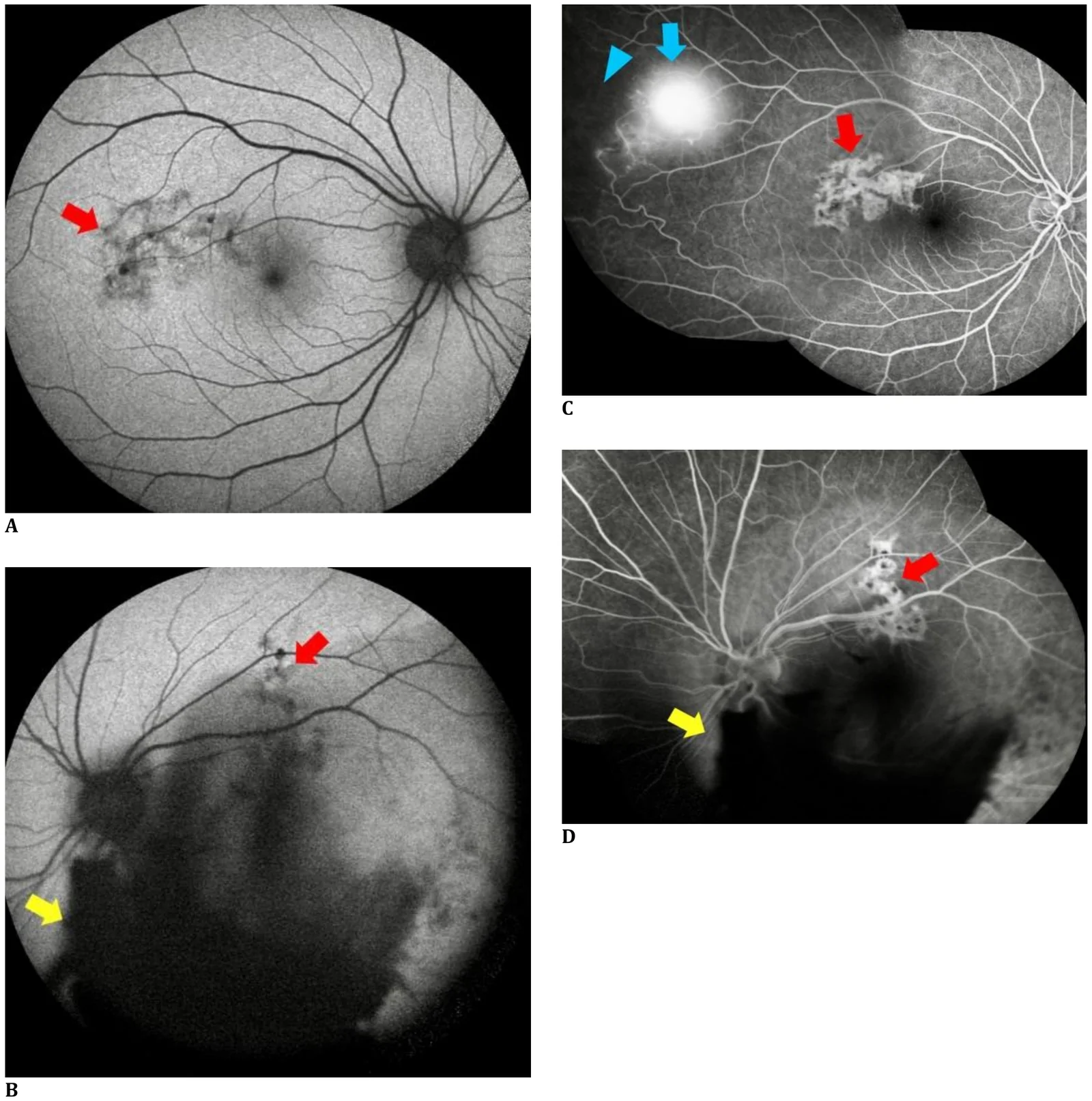
**At presentation.** (**A**) Blue fundus autofluorescence (FAF) imaging of the right eye demonstrating the hypoautofluorescent, placoid areas (red arrow). (**B**) Corresponding FAF image of the left eye showing nearly symmetric hypofluorescent areas, with additional inferior hypofluorescence related to vitreous hemorrhage (yellow arrow). (**C**) Late-phase fluorescein angiography (FA) of the right eye revealing the hyperfluorescence of the placoid lesions consistent with staining (red arrow), retinal neovascularization (blue arrow), increased vascular tortuosity, and peripheral capillary nonperfusion in the temporal periphery (blue arrowhead). (**D**) Corresponding FA image of the left eye demonstrating blocked fluorescence secondary to vitreous hemorrhage (yellow arrow), hyperfluorescence of the placoid lesions (red arrow), and areas of capillary nonperfusion in the temporal periphery

The patient underwent a comprehensive systemic work-up, including a full infectious panel, complete blood count, erythrocyte sedimentation rate, routine biochemistry, and C-reactive protein, all of which were unremarkable. Only the QuantiFERON test was positive. The patient was referred to a pulmonologist to evaluate possible pulmonary involvement of tuberculosis. Though computed tomography of the chest revealed a suspicious-looking infiltrative lesion in the posterior segment of the right upper lobe, mycobacterial culture and polymerase chain reaction testing for *M. tuberculosis* were negative.

Initially, an intravitreal injection of 0.5 mg ranibizumab (Lucentis^®^, Novartis, Basel, Switzerland) was administered into the left eye. Following multidisciplinary assessment, systemic anti-tuberculosis therapy or corticosteroid treatment was not initiated as there was no confirmed active systemic tuberculosis or active choroiditis.

Five weeks after presentation, best-corrected visual acuity was 20/20 in the right eye and 20/30 in the left eye. Vitreous hemorrhage had markedly regressed in the left eye, with only residual inferior vitreous hemorrhage remaining. Ultra-widefield optical coherence tomography angiography (BMizar^®^, TowardPi Medical Technology Ltd., Beijing, China) showed areas of flow deficit in the temporal peripheral retina at the superficial capillary plexus level in the right eye, along with irregular vascular architecture and focal flow voids (**Fig. 2A**). In the left eye, similar irregular vascular architecture and focal flow voids were observed at the superficial capillary plexus level in the temporal retina, accompanied by signal artifacts secondary to residual vitreous hemorrhage (**Fig. 2B**). Sectorial laser photocoagulation was administered all over ischemic areas in both eyes, with special care for avoiding the fibrous ridge present in the left eye.

**Fig. 2:**
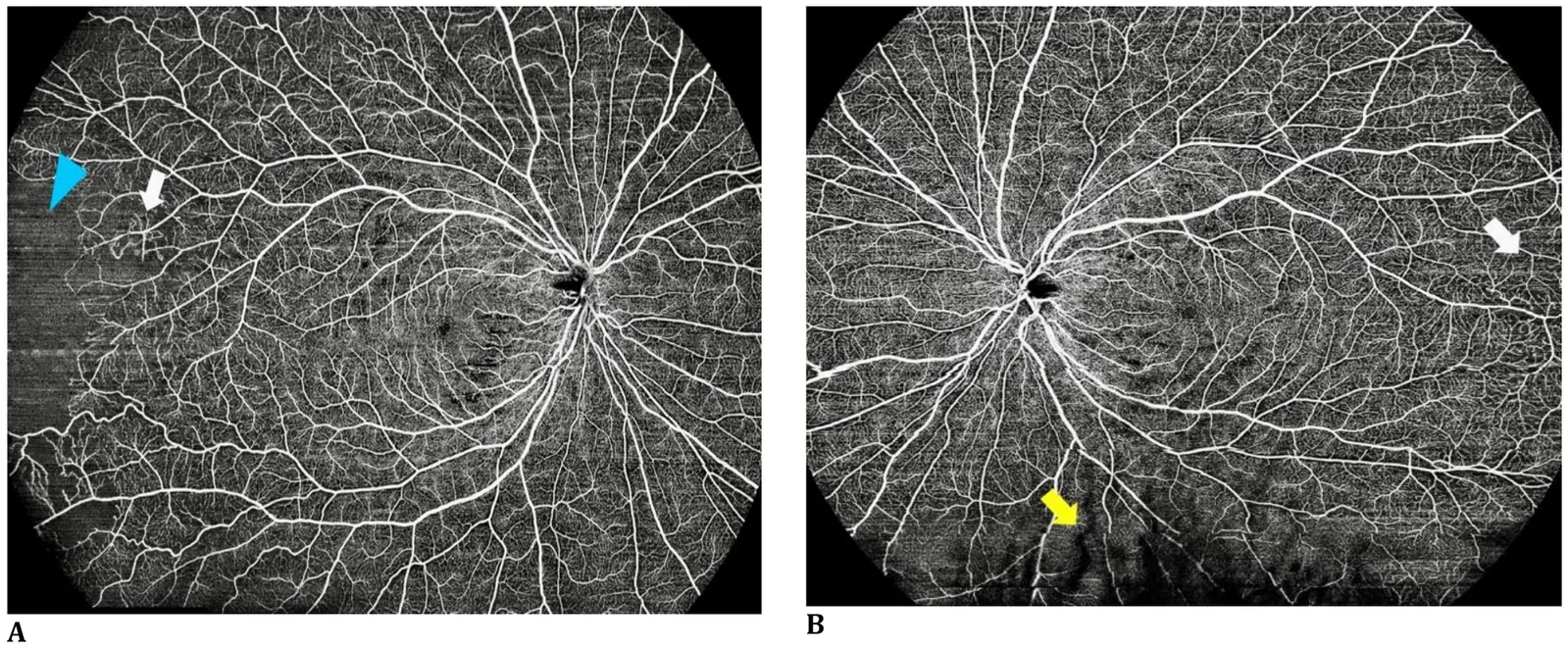
**Five-week follow-up.** Ultra-widefield optical coherence tomography angiography findings at the level of the superficial capillary plexus. Right eye (**A**) demonstrates flow-deficit areas (blue arrowhead), along with irregular vascular architecture and focal flow voids associated with ischemia and retinal neovascularization (white arrow). Left eye (**B**) demonstrates similar irregular vascular architecture and focal flow voids suggestive of ischemia at the superficial capillary plexus level in the temporal retina (white arrow), with signal attenuation artifacts attributable to residual inferior vitreous hemorrhage (yellow arrow)

Two weeks after laser photocoagulation, the patient was asymptomatic. Green fundus autofluorescence imaging (Mirante, Nidek Co., Ltd., Gamagori, Japan) revealed hypoautofluorescent placoid chorioretinal lesions in both eyes, consistent with inactive SLC (**Fig. 3A, B**). Fundus findings were stable in both eyes, with further reduction in vitreous hemorrhage in the left eye (**Fig. 3C, D**).

**Fig. 3:**
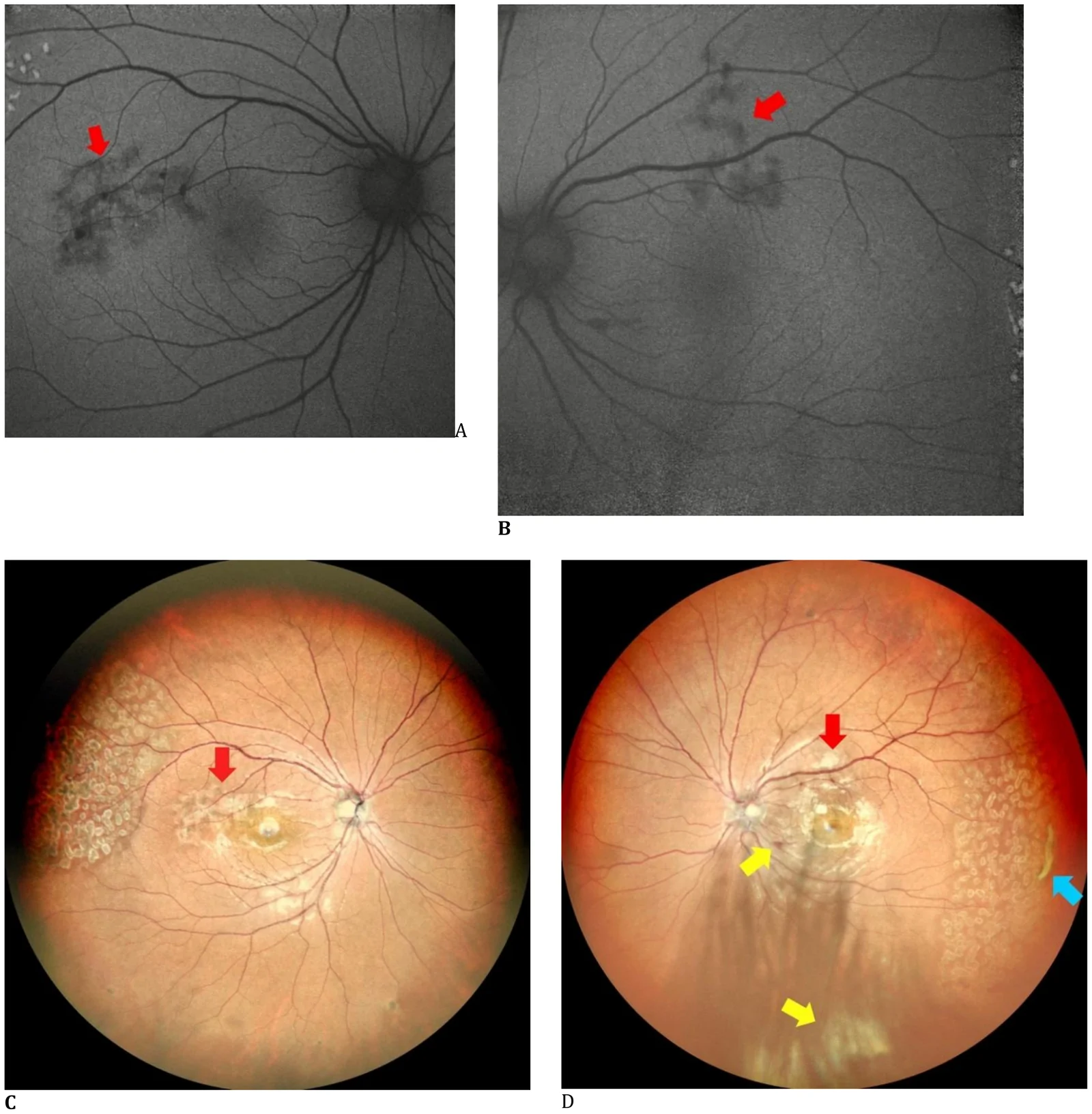
**Seven-week follow-up.** Green fundus autofluorescence images of the right (**A**) and left (**B**) eyes demonstrating the placoid hypofluorescent areas (red arrows). Color fundus photographs (Mirante; Nidek Co., Ltd., Gamagori, Japan) of the right (**C**) and left (**D**) eyes demonstrating the inactive serpiginous-like choroiditis lesions (red arrows), regressed residual vitreous hemorrhage (yellow arrows), a vertical fibrotic ridge (blue arrow), and chorioretinal laser photocoagulation scars in the temporal peripheral retina

## Discussion

This case demonstrates the coexistence of retinal occlusive vasculopathy consistent with Eales’ disease and placoid choroidal inflammation compatible with SLC in a young immunocompetent woman with a positive interferon-gamma release assay in the absence of clinically evident active systemic tuberculosis. The concurrent retinal periphlebitis with peripheral ischemia and choroidal inflammation suggests shared immunopathogenic pathways, indicating that these two entities may represent distinct clinical expressions of the same underlying inflammatory process.

To the best of our knowledge, only two previous reports have documented the coexistence of Eales’ disease and serpiginous-spectrum choroiditis in a single patient. In the first report by Wong et al. [6], a 35-year-old man presented with bilateral mild nongranulomatous anterior uveitis, vitritis, and gray placoid lesions located deep in the retina, consistent with ampiginous chorioretinopathy in both eyes. Although the Mantoux test was positive, no evidence of active systemic tuberculosis was identified. At the four-month follow-up, the patient subsequently developed periphlebitis and intraretinal hemorrhages in the inferonasal quadrant of the right eye, without evidence of retinal neovascularization, findings consistent with Eales’ disease. He was treated with systemic anti-tuberculosis therapy and panretinal laser photocoagulation. Similarly, in our case, SLC lesions were clinically inactive at presentation, suggesting that choroidal inflammation may have preceded or occurred independently of the retinal vasculopathy. However, our case exhibited no anterior segment inflammation or vitritis. She did not receive systemic anti-tuberculosis therapy despite a positive QuantiFERON test, given the absence of active systemic tuberculosis and active choroiditis. Management consisted of intravitreal anti-vascular endothelial growth factor (anti-VEGF) injection and laser photocoagulation of the peripheral ischemic areas.

In the second report, Fung et al. [7] described a 49-year-old man initially diagnosed with Eales’ disease in his left eye, characterized by temporal retinal vascular occlusive disease, arteriolar and venous sheathing, and peripheral retinal ischemia with neovascularization. Given negative QuantiFERON and tuberculin test results and a normal chest radiograph, he was treated with scatter laser photocoagulation, systemic corticosteroids, and mycophenolate mofetil. Nine months later, the patient developed lobular peripapillary choroiditis and peripheral temporal retinal vascular occlusive disease with neovascularization in the right eye, leading to the diagnosis of bilateral Eales’ disease and unilateral right serpiginous choroiditis. Given that his visual acuity remained 20/20, management was limited to laser photocoagulation of the ischemic areas. Over a seven-year follow-up period, progressive bilateral progression of capillary closure and neovascularization were observed, accompanied by cataract, rubeotic glaucoma, cystoid macular edema, and progressive serpiginous choroiditis with vitritis in the right eye. The patient underwent bilateral intravitreal bevacizumab and triamcinolone acetonide injections, right intravitreal dexamethasone implant and peribulbar triamcinolone injections, phacoemulsification with intraocular lens implantation, and pars plana vitrectomy. Polymerase chain reaction analysis of vitreous for *M. tuberculosis* was negative. The authors proposed that the two entities might represent distinct manifestations of a common inflammatory pathway rather than independent tuberculosis-driven conditions. In contrast, the bilateral multifocal placoid lesions in our patient were more consistent with SLC than with the classic course of serpiginous choroiditis, and vitritis was not observed during follow-up. Moreover, immunological evidence of tuberculosis exposure suggests that the observed overlap may more plausibly reflect two phenotypic expressions within the spectrum of tuberculosis-associated ocular inflammation.

Systemic anti-tuberculosis therapy is generally recommended for tuberculosis-related intraocular inflammation, typically in combination with systemic corticosteroids and, when indicated, immunomodulatory drugs [8,9]. In our patient, we did not initially start systemic anti-tuberculosis therapy or corticosteroids because there was no active systemic disease or active choroidal inflammation. Additionally, potential systemic adverse effects were carefully considered as the patient was overweight. Our case underscores the need for individualized therapeutic decision-making in managing tuberculosis-associated ocular inflammatory disorders.

Clinically, the present case also underscores the importance of multimodal imaging in patients with presumed tuberculosis-associated ocular diseases. Peripheral retinal ischemia may be overlooked without widefield angiography [10], whereas subtle SLC lesions may be underestimated without fundus autofluorescence and fluorescein angiography [4]. Furthermore, emerging widefield optical coherence tomography angiography may serve as a valuable adjunctive modality [11], as demonstrated in our case, for assessing the extent of peripheral retinal ischemia.

## Conclusion

In conclusion, this case expands the spectrum of tuberculosis-associated ocular inflammation by demonstrating the concurrent presence of Eales’ disease and SLC in a single patient. Considering the established clinical and pathogenetic differences among ampiginous choroidopathy, serpiginous choroiditis, and SLC, this report appears to represent the first documented coexistence of Eales’ disease and SLC in the same individual. Recognition of such overlap patterns is essential for accurate diagnosis, appropriate monitoring, and individualized treatment strategies.
